# Fractionation of Oligosaccharide Nucleoside Mixtures by Single Pass Nano‐Diafiltration

**DOI:** 10.1002/elsc.70055

**Published:** 2025-10-13

**Authors:** Ulrich Thiele, Tobias Kaloghlian, Jonas Wohlgemuth, Gerald Brenner‐Weiß, André Tschöpe, Matthias Franzreb, Katharina Bleher

**Affiliations:** ^1^ Institute of Functional Interfaces Karlsruhe Institute of Technology Eggenstein‐Leopoldshafen Germany

**Keywords:** 3D‐printing, continuous, glycan, nanofiltration, SPTFF

## Abstract

Glycans, a diverse group of complex oligosaccharides, are critical to human physiology and hold significant potential in medical applications and as food additives. However, their synthesis by glycosyltransferases produces intricate mixtures comprising saccharides, nucleosides, and reaction buffer components, posing substantial challenges for downstream processing and purification. This study aims to establish a continuous, single‐pass nanofiltration process for purifying oligosaccharide‐nucleoside mixtures using a novel dual‐membrane module. We investigated the influence of a static mixer, along with varying flow rates for both the diafiltration and feed streams, on the recovery rate and purity of the final product. Measurements using ESI‐MS assessed product recovery and purity, while buffer removal was monitored through online conductivity measurement. The results demonstrate that incorporating a static mixer nearly doubled the saccharide recovery rate, achieving product purities exceeding 99% and 95%, along with high product recovery rates. Additionally, the reaction buffer system was found to significantly impact the overall process performance. These findings suggest that our novel dual‐membrane module can be effectively utilized for the purification of enzymatically synthesized glycan products.

**Summary**

- The application of a novel 3D‐printed double‐membrane nanofiltration module for the purification of saccharides from enzymatic reaction mixtures was reported in this study.
- In a first step, a suitable nanofiltration membrane was characterized.
- Low recovery rates after single‐pass nano‐diafiltration in the 3D‐printed module were overcome by the application of a static mixer, which highly increased product recovery.
- The notably low purities at high diavolume ratios were traced back to the buffer system, and with an investigation of the influence of different buffer systems on the separation process, we were able to achieve high product purities.
- The results are an important contribution to designing novel purification processes for mixtures resulting from continuous enzymatic synthesis.

**Abbreviations**
FGE: fluid guiding element
FIA‐ESI‐MS: flow injection analysis‐electrospray ionisation‐mass spectrometry
MWCO: molecular weight cut‐off
SPNDFD: single‐pass nanofiltration/diafiltration
SPTFF: single‐pass tangential flow filtration
TFF: tangential flow filtration
UF/DF: ultrafiltration/diafiltration

## 1 Introduction

Glycans are a class of compounds consisting of a diverse array of complex structures made up of glycosidically‐linked monosaccharides. These carbohydrates play a crucial role in human health and are mostly synthesized in the Golgi apparatus, starting from simple linear saccharide chains up to complex branched structures [1, 2, 3]. Expressed and presented on the cell surface, these molecules have a role in immune responses as well as a diverse array of intra‐ and extracellular processes [4, 5]. The tetrasaccharide sialyl‐Lewis^X^, for example, is believed to influence the phenotype of cancer cells [6] and play a role in the human fertilization process [7]. Of high interest are also glycans in the human milk, as they play a vital role in the development of an infant's microbiome and have an impact on the immune system as well as brain development [8, 9].

Due to their mentioned properties, synthesis and purification of glycans are of great interest to the food and pharmaceutical industries [10, 11]. Recombinantly expressed Leloir glycosyltransferases provide a highly regio‐ and stereoselective toolbox for the precise synthesis of complex glycan structures [12]. These enzymes are able to catalyze the transfer of a monosaccharide from a nucleotide‐sugar onto an acceptor‐sugar, building large oligosaccharide structures block‐by‐block. The remaining nucleotide is usually broken down into the nucleoside by an alkaline phosphatase (Figure S1) [13]. Despite small‐scale synthesis being widely established [14], technological applications still present a challenge [15].

Glycosyltransferase reactions result in a complex mixture of sugars, nucleosides, and buffer molecules, which provide a challenge to the efficient recovery of the target sugars. Preparative chromatography is commonly used to purify oligosaccharides both in the lab‐scale as well as for industrial processes [16], such as in the purification of 3’‐sialyllactose [17] as well as mixtures of galactooligosaccharides and fructooligosaccharides [18, 19]. However, chromatography has the disadvantage that it is usually carried out in batches [15], and while simulated‐moving bed chromatography allows for a continuous chromatography process, it is expensive and only cost‐effective for large‐scale processes [16, 20].

Nanofiltration provides a cost‐effective alternative with low energy consumption and a simple operation concept, making it a promising method for the purification of oligosaccharides [21, 22], especially of enzymatically synthesized glycans. It has mainly been applied to the purification of galactooligosaccharides [21, 23] and fructooligosaccharides [24] mixtures, as well as for the separation of small monosaccharides such as xylose and glucose [25]. Discontinuous diafiltration has previously been demonstrated as an effective strategy for purifying enzymatically synthesized human milk oligosaccharide 3′‐sialyllactose from a highly concentrated lactose mixture [26], while continuous diafiltration has been successfully applied for the purification of lactulose syrup [27].

**FIGURE 1:**
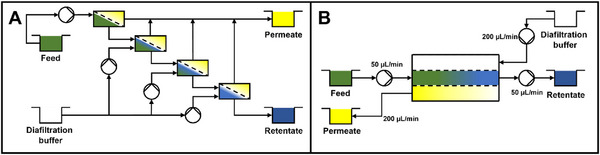
Comparison of the process scheme of a four‐stage continuous nanofiltration/diafiltration process (A) with the proposed single‐pass nanofiltration/diafiltration process (B). The two‐membrane configuration allows for controlled influx of a multiple of the feed volume flow rate through the diafiltration medium stream, effectively consolidating the functionality of a multi‐stage system into a single‐passage module, with—in the shown case—a diavolume of four.

Nanofiltration membranes can be employed in a conventional tangential flow filtration (TFF) system to facilitate the removal of smaller by‐products while maintaining a high concentration of the target oligosaccharide. This approach is analogous to ultrafiltration processes used for biomacromolecule separation [28, 29]. However, a single pass through the system typically results in only a modest improvement in product purity. To enhance purity, either recirculation of the solution in a closed‐loop system or the integration of multiple sequential single‐pass tangential flow filtration (SPTFF) modules can be utilized. Given that a portion of the solution is removed as permeate, the introduction of fresh buffer or ultrapure water—either continuously or in a stepwise manner—is necessary to prevent excessive concentration of the target molecule, akin to diafiltration [30]. While one‐stage TFF requires multiple recirculation rounds [31], a continuous multi‐stage system requires a complex setup to ensure proper sequential concentration and dilution of the mixture (see Figure 1A) [32].

Recently, a novel single‐pass filtration module incorporating two membranes has been developed for continuous ultrafiltration and diafiltration of proteins, demonstrating high‐efficiency buffer exchange [33, 34]. In the present study, we extend the application of this module to nanofiltration, targeting the purification of model mixtures comprising saccharides and the nucleoside uridine. By employing this dual‐membrane system, we can, by setting a diafiltration buffer flow rate that is a multiple of the flow rate of the feed solution, continuously and within a single passage through the module, match several sequential diafiltration cycles (Figure 1B). We systematically evaluate the module's performance with respect to purification efficiency, product recovery, and the effective removal of buffer components usually present in enzymatic synthesis.

## 2 Materials and Methods

### 2.1 Chemicals and Membrane

Unless otherwise stated, all chemicals used in this work were purchased from VWR or Sigma‐Aldrich/Merck and stored according to the manufacturer's instructions and were used without further purification. Analytical experiments were performed using solvents with LC‐MS Grade. All solutions were prepared with ultrapure water (Milli‐Q Gradient, Merck Millipore, Burlington, USA).

The Synder Filtration NFW flat sheet membrane (Table 1) used in this study was purchased from Sterlitech Corporation (Auburn, WA, USA).

**TABLE 1:** Specification of PA‐TFC membrane used in this study according to the manufacturer.

Parameter	Value
Flux (LMH)	76.5–85.0
Filtration area CF‐module (mm^2^)	
*Single‐pass TFF module*	207
*Single‐pass NF/DF module*	189
pH resistance	4–10
Molecular weight cut‐off (Da)	300–500
MgSO_4_ rejection	97.0%
Lactose rejection	98.5%
Polymer material	Polyamide‐TFC

### 2.2 3D‐Printed Nanofiltration Modules

Nanofiltration experiments were performed with self‐designed modules based on the diafiltration modules introduced by Tan et al. [33, 34, 35]. Modules were manufactured by 3D printing with a PolyJet Objet260 Connex3 (Stratasys, Eden Prairie, USA) using VeroClear material. When the printing process was completed, support material was removed manually from the accessible surfaces. Support material inside internal channels was removed by immersing the module parts in 2 M NaOH solution for 24 to 48 h, followed by thorough rinsing with water.

**FIGURE 2:**
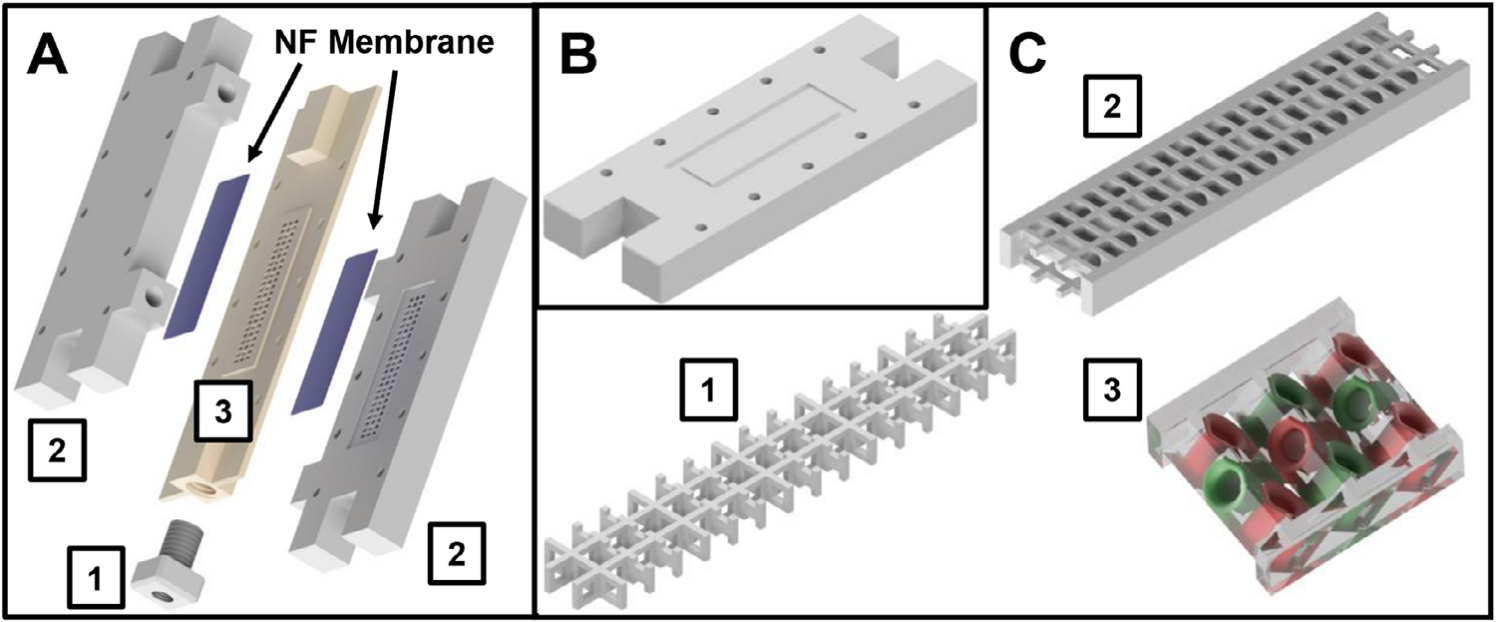
Design of a 3D‐printed module for single‐pass nano‐diafiltration. (A) Assembly drawing of the module parts—1: 3D‐printed adapter for FPLC capillaries; 2: lateral parts with connectors for diafiltration buffer inlet or permeate outlet; 3: middle compartment with hollow‐carved grid structure for membrane support and feed passage. (B) Lateral part for single‐pass classical tangential flow filtration experiments. (C) Different grid structures for the middle compartment—1: standard grid structure used for tangential flow filtration experiments and preliminary single‐pass diafiltration experiments; 2: fluid guiding element used for intensification of single‐pass diafiltration experiments; 3: detailed view of a fluid guiding segment in C‐2.

The nanofiltration module consists of three parts. A middle compartment, through which the feed mixture flows, is enclosed by two nanofiltration membranes. Nanofiltration membranes are fixed by two identical lateral parts (Figure 2A). The assembled module is held together by ten 40 mm M3 screws. In order to perform classical single‐pass tangential flow filtration to characterize single membranes, a separate lateral part consisting of an impermeable side wall was manufactured (Figure 2B).

Middle compartments with two different hollow‐carved structures for membrane support and feed passage were manufactured (Figure 2C). The influence of a standard grid structure (Figure 2C‐1) as well as a stationary mixer (Figure 2C‐2/3), which is hereinafter referred to as a fluid guiding element (FGE), was investigated.

### 2.3 Analytical Experiments

Analytical measurements were performed via flow injection analysis‐electrospray ionization‐mass spectrometry (FIA‐ESI‐MS) as previously described [36] on a TripleTof 6600+ mass spectrometer from SCIEX (AB SCIEX LLC, Framingham, USA) equipped with a SCIEX M5 Micro LC‐TE (AB SCIEX LLC, Framingham, USA) for high‐throughput sample measurement. The M5 Micro LC‐TE enables direct injection for FIA measurements. The mass spectrometer was calibrated using NEG PPG 3e^−4^ M solution (AB SCIEX LLC, Framingham, USA) with the instrument optimization function of the Analyst Software (version 1.8.1, AB SCIEX LLC, Framingham, USA) prior to measurement.

Samples were diluted to a final concentration of 1:50,000 in 50:50 ACN/H_2_O and spiked with 100 ng 1–^13^C Glucose. 6 µL of the sample volume was analyzed via direct injection measurements with a flow rate of 50 µL min^−1^ in product ion scan mode with negative polarity. The mass range was set between m/z 60 and 600 Da with an accumulation time of 0.25 s per scan.

Data processing was performed using SCIEX OS Software (version 2.1, AB SCIEX LLC, Framingham, USA) and the smoothing was set to “high.” For quantification, an extracted ion chromatogram (EIC) with a width of ±0.02 was defined. ESI‐Spray (Table S1), fragmentation settings (Table S2), and the calibration curves (Table S3 and Figure S3) for the analytes can be found in the supplementary information.

### 2.4 Filtration Experiments

Single‐pass tangential flow filtration (SPTFF) as well as single‐pass nanofiltration/diafiltration (SPNFDF) experiments were performed with an automated ÄKTA Pure 25 M FPLC setup (Cytiva, Global Life Sciences Solutions USA LLC, Marlborough, MA, USA).

The feed was composed of 5 mM of a respective saccharide and 5 mM uridine in either 100/25 mM HEPES/KCL buffer (pH 7.5), 100 mM MOPS buffer (pH 7.0), or 50/150 mM TRIS/NaCl buffer (pH 7.4). SPTFF experiments were performed with a feed flow rate of 1 mL min^−1^ using the 3D printed module and replacing one of the lateral parts with the alternative version having an impermeable side wall (Figure 2B). Transmembrane pressure (TMP) was generated by passing the outlet flow through a PEEK capillary with an internal diameter of 0.125 mm. A loop valve (V9‐L) was used to switch between four capillaries with increasing length to generate a stepwise increase of the transmembrane pressure (Figure S2). TMP was calculated with the measured pressures directly in front of the inlet and between the outlet and the V9‐L valve. Permeate pressure was assumed to be atmospheric. Permeate samples were fractioned with an F9‐C fraction collector once a constant UV‐signal was reached and subsequently weighed for the determination of the flux and analyzed via FIA‐MS to determine membrane rejection.

The rejection was calculated as follows:

$$R_{i}=1-\frac{c_{i,P}}{c_{i,F}}$$

**FIGURE 3:**
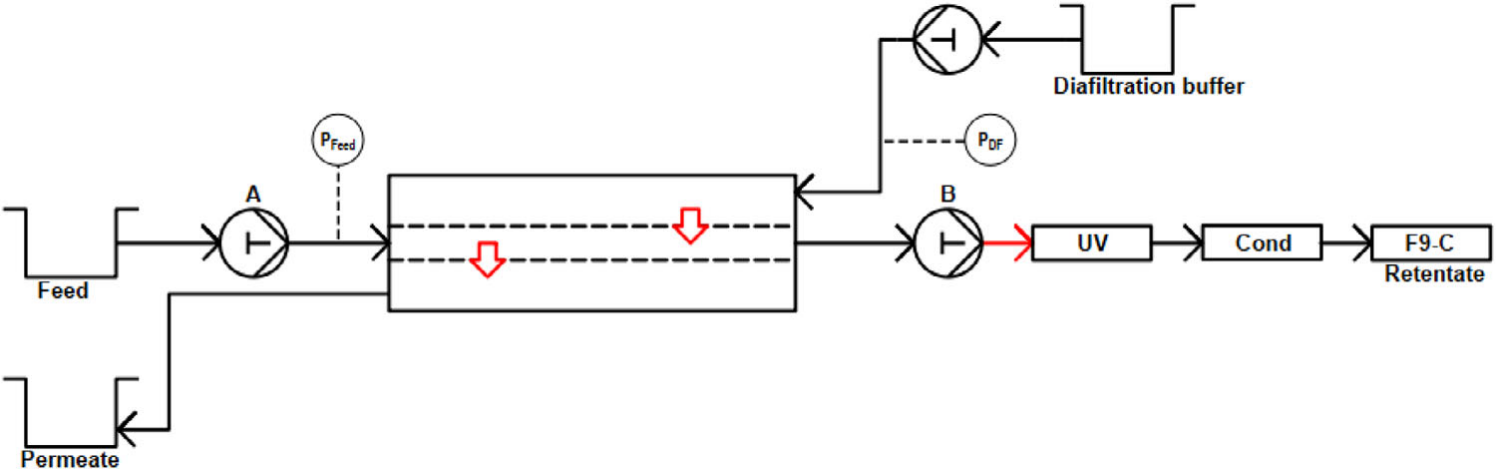
Process scheme of the experimental setup for single‐pass nano‐diafiltration experiments using an ÄKTA Pure 25 M. The 3D printed module was installed between the A and B pumps with the percentage of pump B set to 50% of the total flow (A+B), resulting in equivalent pump rates in the in‐ and outlet of the middle compartment. Salt and nucleoside concentrations were measured in real‐time by the built‐in UV and conductivity sensors of the FPLC system. Pressure in the middle compartment was measured in front of the feed inlet. A UPLC capillary (in red) was used between pump B and the UV sensor to generate 30 bar of backpressure, ensuring a correct flow rate of pump B even in case of increased pressures on the intake side. The diafiltration buffer was pumped by an S9 sample pump with a built‐in pressure measurement device. Retentate samples were fractionated with an F9‐C fraction collector.

For SPNFDF experiments, the module (Figure 1A) was installed between pumps A and B of the ÄKTA FPLC system with a flow rate ratio between A and B set to 1:1 (pump B gradient set at 50%). To ensure proper flow rate in the outlet, a 167 cm UPLC capillary was installed between pump B and the UV‐sensor, generating a backpressure of around 30 bar (Figure 3). A feed flow rate Q_Feed_ of either 100 or 50 µL min^−1^ was set. The diafiltration medium (ultrapure water) was pumped through the external S9 sample pump with a diafiltration flow rate Q_DF_ that was varied in steps of 50 or 100 µL min^−1^ from 0 to 300 µL min^−1^. At each step, Q_DF_ was kept constant until a constant UV‐signal was reached, indicating the process reached a steady‐state. In this stationary state, three samples were fractioned in 10‐min intervals with the F9‐C fraction collector. The buffer concentration was monitored in real‐time with the built‐in conductivity sensor, and saccharide and uridine concentration in the samples was determined via FIA‐MS.

The degree of buffer removal was calculated via conductivity measurements. Calibration curves were established by mixing the buffer with ultrapure water using the gradient function of the ÄKTA system to measure the conductivity of different ratios of buffer system and ultrapure water. The calibration curves can be found in the Figure S4.

## 3 Results and Discussions

### 3.1 Membrane Characteristics

The separation performance of small polarized or ionic compounds by nanofiltration membranes is not only dependent on steric exclusion, but also on the interaction of the compound with the electric charge of the membrane surface, which is described by Donnan exclusion [37, 38]. Polyamide composite membranes, such as the one used in this work, have been shown to be negatively charged at neutral pH [39, 40, 41], and the NFW membrane has been shown to have a negative zeta potential at pH 7.2 [42]. Uridine, having a p*K*_a_ value of 9.25 [43], therefore, should have a higher retention once it is deprotonated at higher pH values.

The membrane used in this study has a MWCO of 300–500 Da and is, according to the manufacturer, especially suitable for the enrichment of lactose (M_w_  =  342.29 g mol^−1^). Taking into account that raffinose and stachyose have molecular weights of 504.42 g mol^−1^ and 666.58 g mol^−1^, respectively, the size exclusion effect should allow for the enrichment of all three saccharides. Uridine with a molecular weight of 244.2 g mol^−1^ should only be partially rejected by the membrane as it is not negatively charged at physiological pH values and has a molecular weight slightly below the MWCO.

**FIGURE 4:**
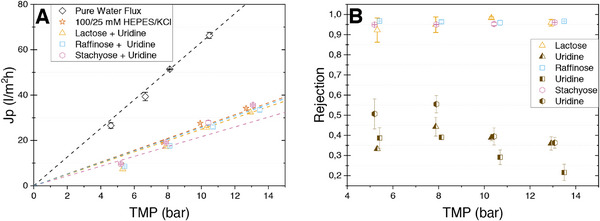
Permeability and rejection properties of the NFW membrane. (A) Permeate flux of pure water, 100/25 mM HEPES/KCl buffer (pH 7.5), as well as model mixtures at different TMP. (B) Rejection of the model product mixtures of either lactose, raffinose, or stachyose with uridine in 100/25 mM HEPES/KCl Buffer (pH 7.5) at different TMP.

At the beginning of the membrane characterization, the measured fluxes were plotted versus the applied TMP (Figure 4A). The slopes of the respective curves correspond to the permeability of the membrane when passed by different solutions. First, the water flux of the membrane was determined by applying ultrapure water. Next, a plain buffer containing 100/25 mM HEPES/KCl at pH 7.5 was fed into the module. Finally, model mixtures of 5 mM of the saccharide of interest and 5 mM uridine in 100/25 mM HEPES/KCl at pH 7.5 were used. Buffer composition and concentration of the analytes were chosen according to common product mixtures in the synthesis of glycans [44, 45].

The permeability of ultrapure water of the membrane was determined at 6.32 ± 0.01 L m^−2^ h^−1^ bar^−1^, which is significantly lower than the manufacturer's specified value of 10.08 L m^−2^ h^−1^ bar^−1^. The pressure drop due to the capillary between permeate outlet and fraction collector, as well as the capillaries between feed inlet/outlet and the pressure sensor, is negligibly small and does not explain the difference between the specification and the measured value. The influence of the support grid on the flux could be more intricate, extending beyond a simple coverage of the membrane surface. Furthermore, variations in the membrane material may partially account for the discrepancies observed in comparison to the data sheet specifications. Nevertheless, the observed water flux is in the correct magnitude and shows that the 3D‐printed module can withstand pressures of more than 12 bar and is suitable to operate under nanofiltration conditions.

The permeabilities for the pure buffer solution HEPES/KCl and mixtures of buffer containing lactose, raffinose, and stachyose with uridine were 2.61 ± 0.14 L m^−2^ h^−1^ bar^−1^, 2.49 ± 0.01 L m^−2^ h^−1^ bar^−1^, 2.16 ± 0.12 L m^−2^ h^−1^ bar^−1^, and 2.56 ± 0.15 L m^−2^ h^−1^ bar^−1^, respectively (Figure 4A). This implies that the permeability of the buffer solutions and the model mixtures is around 2.5 times lower than that of pure water. The considerably negligible difference between the permeability of the four solutions might be due to the high concentration of HEPES present in all four solutions. HEPES has a molecular weight of 238.31 g mol^−1^ and a p*K*_a2_ of 7.48 [47], at which the neutral molecule becomes negatively charged. A combination of partial rejection through steric exclusion and substantial rejection due to the negative charge of HEPES at pH 7.5 may contribute to the observed trend. The rejection of HEPES results in a concentration polarization layer of the buffer at the membrane surface, causing a notably reduced permeability [48, 49].

Figure 4B shows the dependency of the measured retention, expressed as rejection, with regard to the applied TMP. The high rejection for lactose (94.9% at 7.87 bar) only differs by 4% from the manufacturers specification. The high rejection for raffinose and stachyose was also expected due to size exclusion. The rejection of uridine lies between 20% and 55% depending on TMP and the used saccharide. Independent of the saccharide, uridine rejection shows the familiar trend known for nanofiltration, saying rejection increases slightly when raising TMP from 5 to 8 bar [50]. However, when TMP is increased further to 10.5 and 13.5 bar, the trend reverses and uridine rejection drops below the value at 5 bar. The reason for this unusual behavior is not fully understood, however, we assume that it is related to the fact that the molecular weight of uridine is slightly below the MWCO of the membrane and therefore shows a behavior between the one of very small molecules (Mw << MWCO) which simply are carried with the water flux through the membrane and larger molecules (Mw > MWCO) which are retained on top of the membrane. At the beginning, the effect of increased water flux with increasing TMP dominates, resulting in a stronger dilution of uridine in the permeate and consequently higher rejection factors. However, uridine and saccharide rejection result in concentration polarization on top of the membrane. In consequence, the amount of uridine carried with the water flux through larger pores of the membrane strongly increases. At pressures higher than 8 bar, this effect dominates for uridine, having a MW slightly below the MWCO, and results in a reduction of the rejection factor with further increasing TMP. A related effect of increased solute concentrations in the permeate stream with increasing permeate to feed volume ratios has also been observed in dead‐end nanofiltration [51]. In contrast to the behavior of uridine, oligosaccharides are large enough to be retained also by larger pores in the membrane, and their rejection stays high even at TMPs up to 15 bar.

Independent of details of partial uridine rejection, the high difference in the rejection shows that the NFW membrane is well suited to efficiently separate polysaccharides from the nucleoside uridine for the downstream processing after enzymatic glycan synthesis.

### 3.2 Single Pass Nanofiltration Intensification Through the Application of a Fluid Guiding Element

**FIGURE 5:**
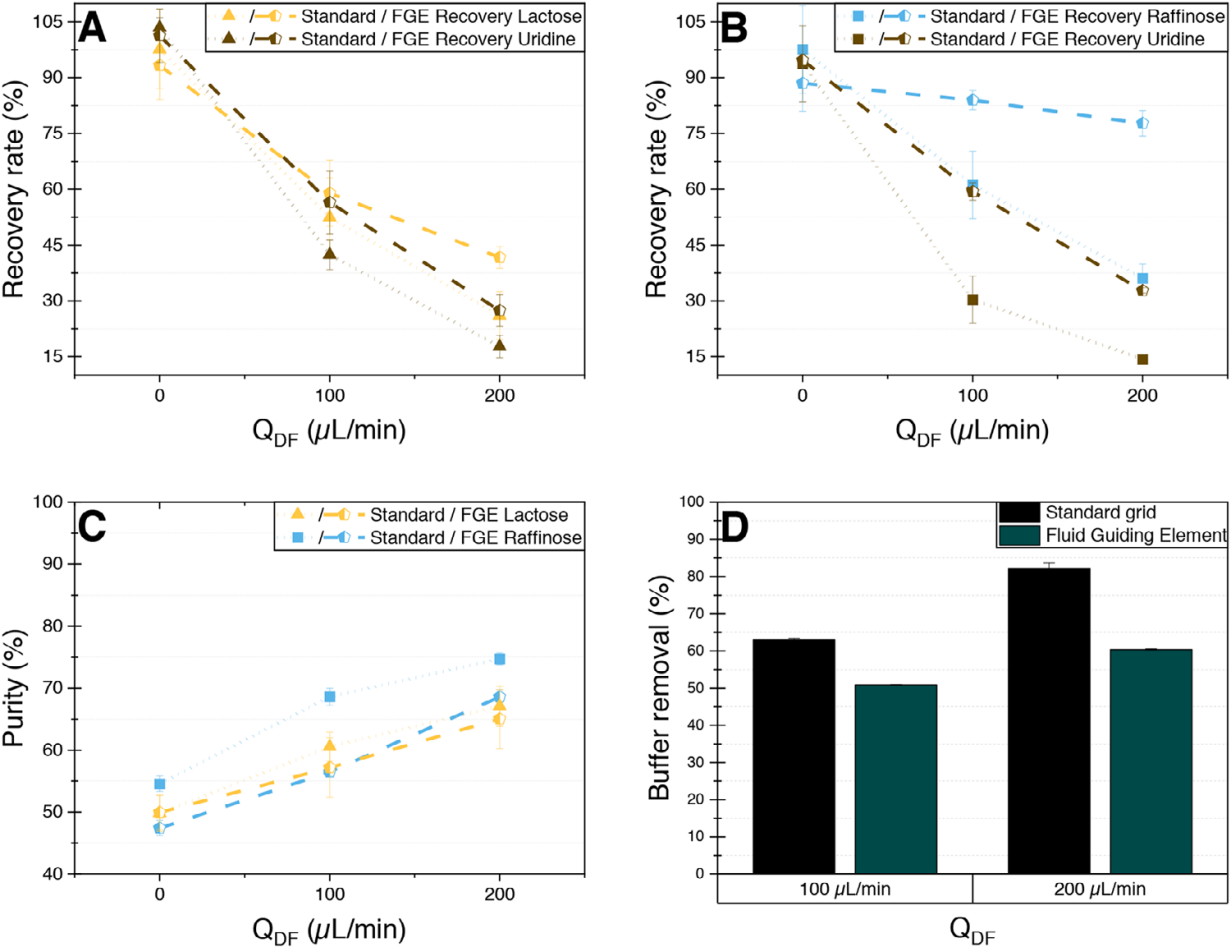
Recovery rate, purity and ratio of buffer removal of the model product mixtures of 5 mM lactose or raffinose and 5 mM uridine in a 100/25 mM HEPES/KCl buffer system at pH 7.5 after single‐pass nanofiltration/diafiltration in a 3D‐printed module with a feed flow rate of 100 µL min^−1^ and an increasing flow rate of the diafiltration medium ultrapure water between 0 and 200 µL min^−1^. (A) Comparison of recovery rates of lactose and uridine from experiments performed with the standard grid structure as well as the fluid guiding element. (B) Comparison of recovery rates of raffinose and uridine from experiments performed with the standard grid structure as well as the fluid guiding element. (C) Comparison of purities of lactose and raffinose from experiments performed with the standard grid structure as well as the fluid guiding element. (D) Comparison of the buffer removal ratio from experiments performed with the standard grid structure as well as the fluid guiding element.

The use of two membranes and applying a transversal diafiltration buffer flow in the single‐pass module can lead to differences in process behavior when compared to classic tangential flow filtration. As a result, even though rejection factors measured in a classical TFF arrangement indicate that the membrane can separate the model mixtures, they are not sufficient to quantitatively predict the performance of the in‐situ purification within the novel SPNFDF module. Therefore, a first series of experiments was conducted to determine purity and recovery rate when applying mixtures of lactose and raffinose with uridine in this module. Mixtures containing 5 mM of either the disaccharide lactose or the trisaccharide raffinose and 5 mM uridine in 100/25 mM HEPES/KCl buffer at pH 7.5 were fed into the module with a flow rate of 100 µL min^−1^. During the runs, the ratio between the transversal flow of the diafiltration buffer and the feed flow (Q_DF_/Q_Feed)_ was successively increased by increasing the diafiltration buffer feed from 0 to 200 µL min^−1^. Experiments conducted with the standard grid structure in the middle compartment (Figure 2C‐1) resulted in a product purity of only 60.5% and 67% for lactose at a Q_DF_/Q_Feed_ ratio of 1 and 2, respectively. The purity of raffinose increased from 54% in the feed mixture to 68.6% and 74.7% at a Q_DF_/Q_Feed_ ratio of 1 and 2, respectively (Figure 5C). At a Q_DF_/Q_Feed_ ratio of 2, nearly half of the uridine was removed from the mixture within a single pass through the module. However, the process resulted in significant losses of the polysaccharide, as only 36% of the raffinose in the feed was recovered at the outlet (Figure 5B). For lactose, a Q_DF_/Q_Feed_ ratio of 2 resulted in an even lower recovery rate of 26% (Figure 5A), which is expected as lactose has a smaller molecular weight. The lower purity of lactose at a diafiltration volume of 2 can be attributed to this higher loss.

These results illustrate the aforementioned challenge when going from a classic tangential flow filtration system toward the combined nanofiltration/diafiltration system, which adds a transversal convective flow of diafiltration buffer, improving the removal of uridine by increasing the permeate flow but also increasing the concentration polarization of retained molecules at the membrane surface. This accumulation can, in turn, lead to an elevated concentration gradient across the membrane, thereby potentially enhancing diffusive transport and increasing saccharide loss. This effect of concentration polarization is added on top of the stronger convective transport because of the increased permeate flow. Given the presumption that a boundary layer enriched with the polysaccharides is contributing to suboptimal yields, enhancing dispersion within the system should reduce this layer, thereby potentially improving yield efficiencies. In consequence, a middle compartment with a 3D‐printed fluid guiding element (FGE) structure, which has been shown to act similarly to a stationary mixer [52, 53], was 3D‐printed and tested with respect to its effect on recovery rate and purity. Experiments utilizing the FGE structure (Figure 2C‐2) were conducted as previously described for the standard grid structure. The results show that while purity remained nearly constant (Figure 5C), the FGE structure resulted in a significant increase in yield for both product mixtures. The recovery rate for lactose was nearly doubled from 26% to 49.5% (Figure 5A). Raffinose recovery even more than doubled from 36% to 77% (Figure 5B). At a Q_DF_/Q_Feed_ ratio of 2, uridine recovery also increases markedly with the use of a fluid guiding element (FGE) compared to a standard grid, increasing from 17% to 27% in lactose mixtures (Figure 5A) and from 14% to 32% in raffinose mixtures (Figure 5B). Concurrently, the ratio of buffer removal decreases from 82% to 60% (Figure 5D), meaning that 40% of the HEPES buffer remains in the module. This retained HEPES may contribute to the formation of a resistance layer, which reduces uridine passage and, consequently, enhances the unwanted recovery. Whether the significantly higher recovery of the saccharide in raffinose mixtures compared to lactose might also contribute to an increased resistance requires further investigation.

In each segment, the FGE structure splits the incoming flow into an upper and lower part. These two are then successively divided into different channels and merged in consecutive chambers. This causes small turbulences or swirls [53], increasing dispersion inside the system as well as disrupting the saccharide concentration layer on the membrane, which in turn reduces diffusive transport through the membrane and increases recovery rates. As a consequence, all the following experiments were conducted using the single‐pass module with FGE.

**FIGURE 6:**
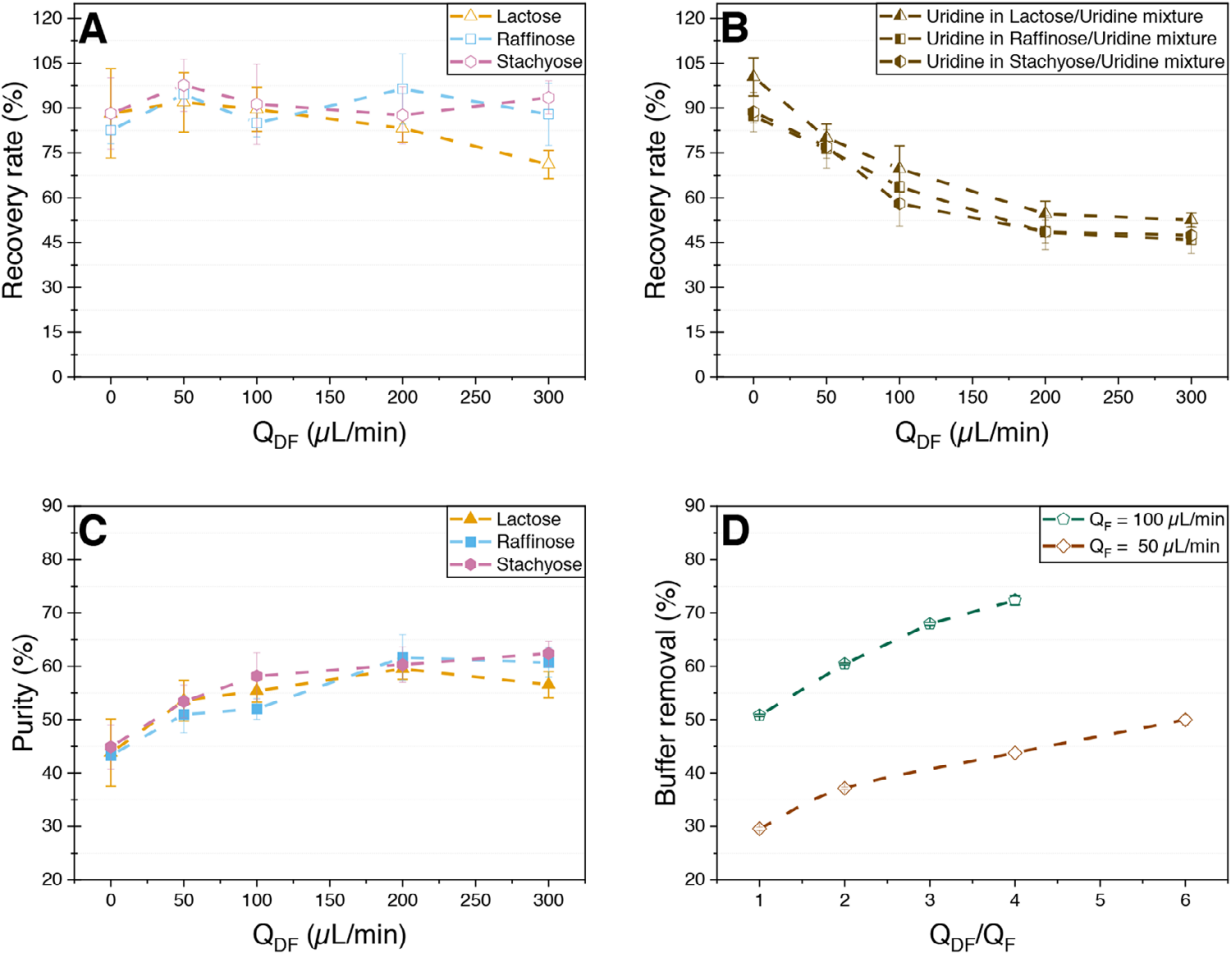
Recovery rate, purity and ratio of buffer removal of the model product mixtures of 5 mM lactose or raffinose and 5 mM uridine in a 100/25 mM HEPES/KCl buffer system at pH 7.5 after single‐pass diafiltration in a 3D‐printed SPNFDF module with a feed flow rate of 50 µL min^−1^ and an increasing flow rate of the diafiltration medium ultrapure water between 0 and 300 µL min^−1^. (A) Recovery rate of the lactose, raffinose, and stachyose. (B) Recovery rate of uridine in saccharide‐uridine mixtures. (C) Purity of lactose, raffinose, and stachyose. (D) Comparison of buffer removal from experiments performed with a fluid guiding element with feed flow rates of 50 and 100 µL min^−1^.

A critical bottleneck for further improving purity is the restricted flux of diafiltration medium through the membrane, attributed to its relatively low permeability. This limitation induces elevated pressures, and operating at flux levels substantially higher than those recommended by the manufacturer, resulting in a risk of membrane damage. To enhance the Q_DF_/Q_Feed_ ratio and determine if higher purities can be achieved, the feed flow rate was reduced to 50 µL min^−1^, while the flow rate of the diafiltration medium was increased up to 300 µL min^−1^, compared to the previous 200 µL min^−1^. This corresponds to a maximum diafiltration volume ratio of 6:1 instead of 2:1. Since higher recovery rates of the saccharides also contribute to better purities and the recovery rate should increase with the molecular weight, the purification of a mixture with 5 mM stachyose was also investigated. The recovery rates for all three saccharides remained consistently similar between 80% and 100%. Notably, it can be observed that at the highest diafiltration volume ratio of 6:1, the recovery rate increased with the molecular weight from 71% for lactose to 96% for stachyose (Figure 6A). The recovery rate for uridine is consistent across all three mixtures, showing no significant variation and appears to reach a saturation point, capping at around 50% (Figure 6B). The increase in purity is significantly lower in comparison to the experiments with a higher feed flow rate, reaching a maximum of around 60% at a Q_DF_/Q_Feed_ of 4 without an increase at a ratio of 6:1 (Figure 6C).

The mixing capability of the fluid guiding element is influenced not only by the ratio of the diafiltration stream to feed but also by the feed flow rate itself, indicating that both parameters are critical for optimizing performance. Conductivity measurements reveal that the buffer removal ratio is significantly lower at a feed flow rate of 50 µL min^−1^ compared to 100 µL min^−1^ across all Q_DF_/Q_Feed_ ratios (Figure 6D), underscoring the substantial effect of feed flow rate on buffer removal efficiency. Lower buffer molecule removal results in a stronger resistance layer due to the negative charges of HEPES, which in turn contributes to higher recovery rates for both saccharides and uridine. From this, we put forward the hypothesis that the observed low purities can be attributed to the interaction between the HEPES buffer and the nanofiltration membrane. Given the negative charge of the NF membrane and the fact that HEPES is negatively charged at pH 7.5 [46, 47], a significant electrostatic repulsion exists between HEPES and the membrane surface. This repulsion surpasses the convective force, resulting in the formation of a HEPES concentration polarization layer. This layer obstructs the passage of other molecules through the membrane, leading to the reduced purities and relatively high yields observed in the experimental results.

In consequence, we shifted the focus to the role of the buffer in which the sugars are dissolved. The following sections explore the formulated hypothesis that the buffer composition has a significant impact on the system's separation efficiency. This analysis is crucial for gaining a deeper understanding of the process parameters as well as choosing a reaction buffer that allows for an effective separation.

### 3.3 Influence of Buffer Solution on Saccharide Purity and Buffer Exchange Ratio

To gather additional evidence supporting the aforementioned hypothesis regarding the unwanted effects of an accumulated HEPES layer at the membrane surface, mixtures of 5 mM raffinose and 5 mM uridine in different buffer systems, but at comparable pH, were screened. Buffer solutions of 100 mM MOPS buffer at pH 7.5 and 50/150‐mM TRIS/NaCl buffer at pH 7.4 were tested, as glycosyltransferases have been shown to be active in both systems [54, 55]. Compared to HEPES, MOPS, and TRIS, both have lower molecular weights (209.3 g mol^−1^ and 121.1 g mol^−1^) and p*K*_a_ values of 7.2 and 8.0, respectively [56], which should, in both cases, reduce the tendency to form a concentration polarization layer. Furthermore, the 100/25 mM HEPES/KCl solution was diluted with water in a proportion of 1:10, yielding a 10/2.5 mM buffer solution. Experiments were conducted with a feed flow rate of 50 µL min^−1^.

**FIGURE 7:**
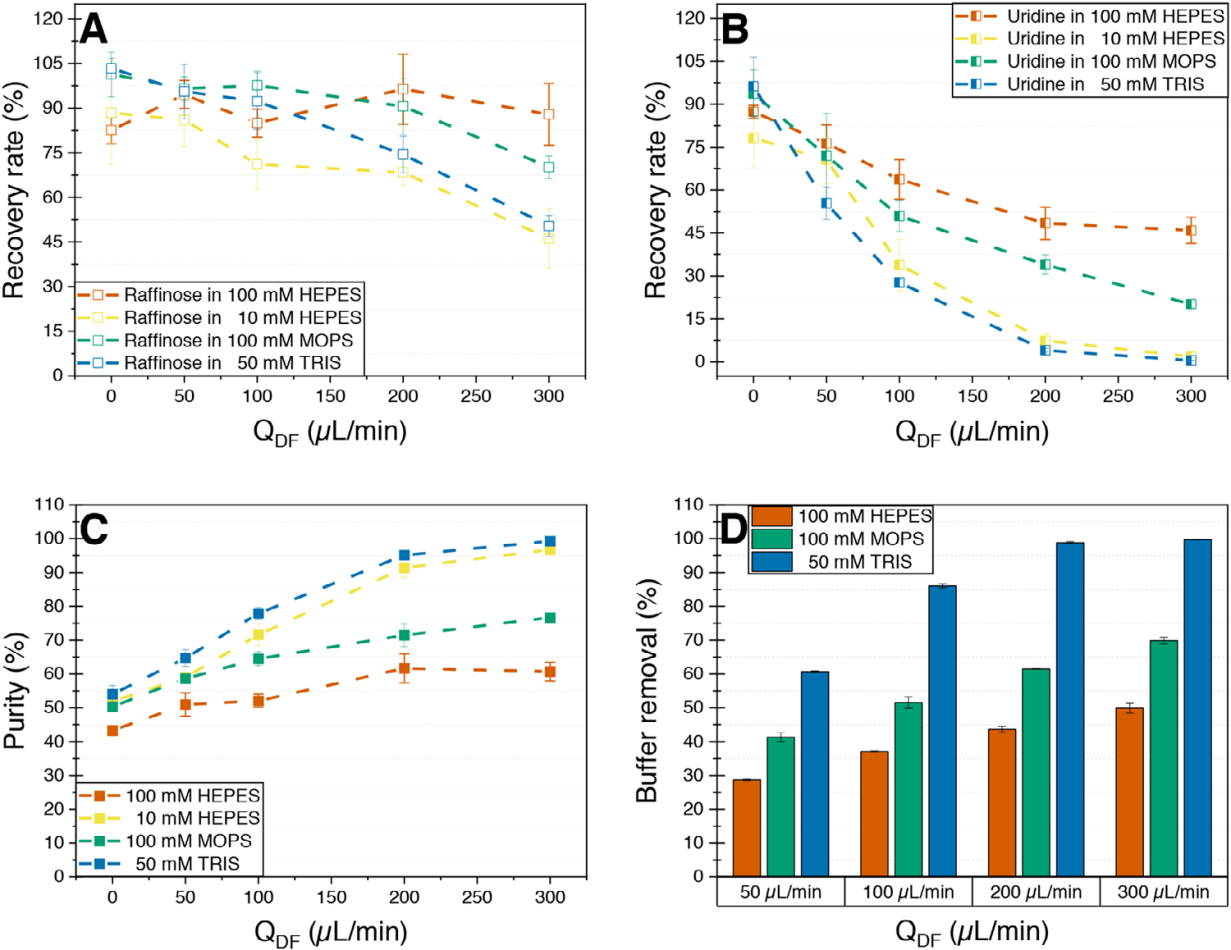
Recovery rate, purity and ratio of buffer removal of the model mixture of 5 mM raffinose and 5 mM uridine in 100/25 mM HEPES/KCl (pH 7.5), 10/2.5 mM HEPES/KCl (pH 7.5), 100 mM MOPS (pH 7.2) and 50/150 mM TRIS/NaCl (pH 7.4) after single‐pass nanofiltration/diafiltration in a 3D‐printed module with a feed flow rate of 50 µL min^−1^ and an increasing flow rate of the diafiltration medium ultrapure water between 0 and 300 µL min^−1^. (A) Recovery rate of raffinose from experiments performed in the four different buffer systems. (B) Recovery rate of uridine from experiments performed in the four different buffer systems. (C) Purity of raffinose from experiments performed in the four different buffer systems. (D) Degree of buffer removal for the HEPES, MOPS, and TRIS buffer systems.

The achieved product purity at the highest ratio of Q_DF_/Q_Feed_ of 6:1 is 77.1% for the solution of raffinose/uridine in 100 mM MOPS buffer compared to 58.7% in 100 mM HEPES, which is an increase of 18.4% (Figure 7C). The optimum for MOPS can be found at Q_DF_ of 200 µL min^−1^, where a purity of 74.5% was achieved at a yield of 90.6%. This demonstrates a significant improvement compared to 100 mM HEPES without a substantial loss of product (Figure 7A).

An even better product purity of 91.4% and 96.7% was achieved in a 10 mM HEPES buffer at Q_DF_/Q_Feed_ ratios of 4:1 and 6:1, with corresponding recovery rates of 68.4% and 46.1%, respectively (Figure 7A). These results represent a significant improvement in purity compared to those obtained with 100 mM HEPES and MOPS buffers, highlighting the detrimental impact of high HEPES concentrations on the purification process. Additionally, the reduced yield of 68.4% (compared to 96.3% in 100 mM HEPES) at a Q_DF_/Q_Feed_ ratio of 4:1 provides further evidence of an additional resistance layer in the case of high HEPES concentrations that obstructs the passage of both raffinose and uridine. The best combinations of high purities and yields were obtained in a 50/150 mM TRIS/NaCl buffer at pH 7.4. Specifically, a raffinose purity of 95.1% was achieved with a yield of 74.4% at a Q_DF_/Q_Feed_ ratio of 4:1. At a higher Q_DF_/Q_Feed_ ratio of 6:1, the purity increased to 99.2%, albeit with a substantial yield reduction to 49.6% (Figure 7C).

The removal of uridine is strongly influenced by the type and concentration of buffer used in the mixture. In a HEPES‐buffered system, the recovery rate of uridine decreases markedly from 48% to 7.4% at a Q_DF_/Q_Feed_ ratio of 4 when the HEPES concentration is reduced from 100 mM to 10 mM, corresponding to a 78% increase in uridine removal. Optimal uridine removal was achieved in a 50/150 mM TRIS/NaCl (pH 7.4) buffer, where only 4% of uridine was recovered at a Q_DF_/Q_Feed_ ratio of 4, further decreasing to 0.4% at a ratio of 6.

The results for the removal of buffer molecules follow the pattern observed for uridine, with higher degrees of buffer removal achieved for MOPS and TRIS compared to HEPES (Figure 7D). Specifically, 98.8% of 50/150 mM TRIS/NaCl was removed at a Q_DF_/Q_Feed_ ratio of 4:1, while only 43.6% 100/25 mM HEPES/KCl could be removed at the same ratio. The removal of HEPES at Q_DF_/Q_Feed_ ratio of 6:1 reaches 49.8%. Potassium chloride has a molecular weight of 74.5 g mol^−1^, meaning that it most likely contributes mostly to the conductivity decrease observed for the HEPES/KCl buffer system. Therefore, even more than 50.2% of HEPES remains in the middle compartment of the module. Previous experiments also showed that a 100/25 mM HEPES/KCl solution (pH 7.5) has a similar conductivity (3 mS cm^−1^) as the product mixture gained with the HEPES buffer (3.6 mS cm^−1^).

**FIGURE 8:**
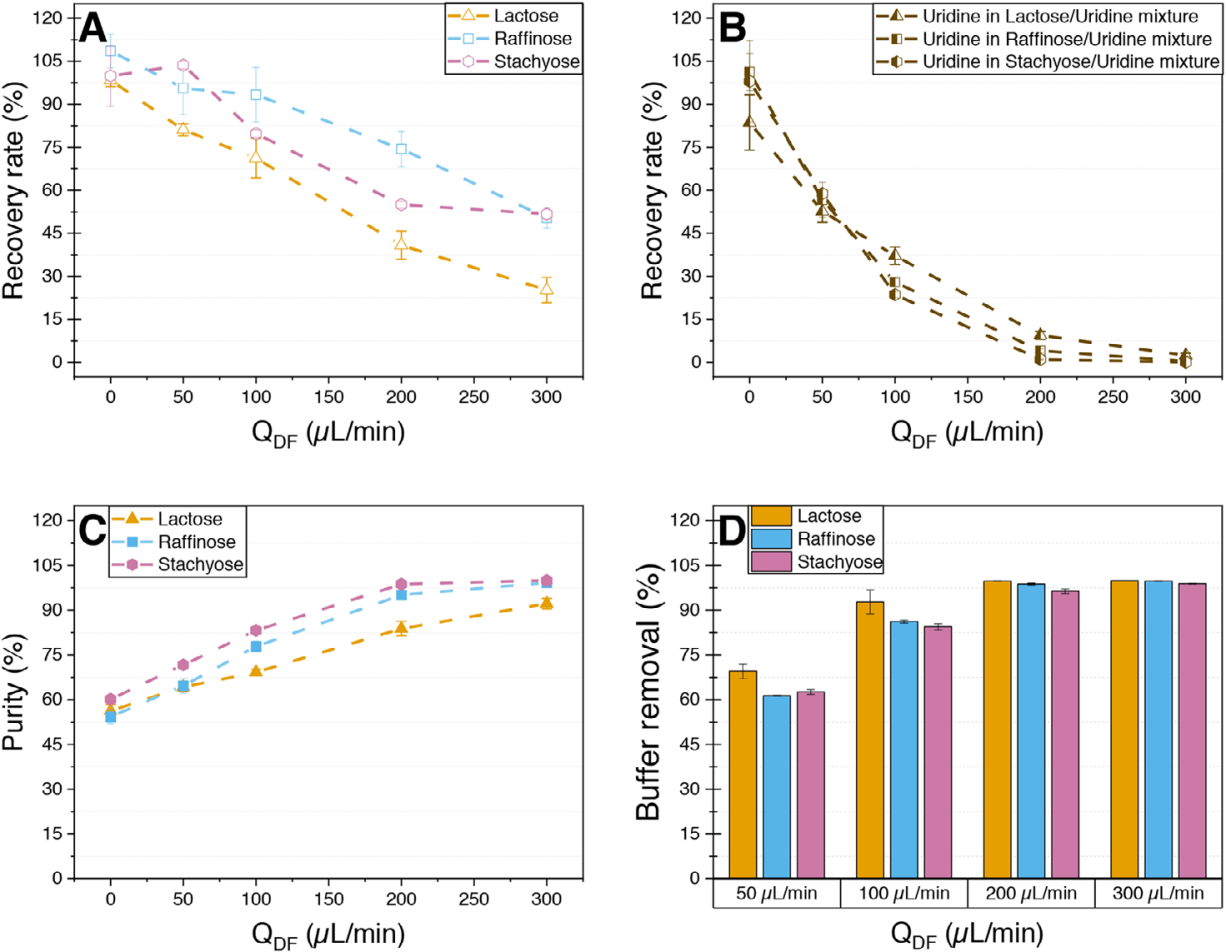
Recovery rate (A+B), purity (C) and ratio of buffer removal (D) of the model product mixtures of 5 mM lactose, raffinose or stachyose with 5 mM uridine in a 50/150 mM TRIS/NaCl buffer system at pH 7.4 after SPNFDF with a feed flow rate of 50 µL min^−1^ and an increasing flow rate of the diafiltration medium between 0 and 300 µL min^−1^. (A) Recovery rate of lactose, raffinose, and stachyose. (B) Recovery rate of uridine. (C) Purity of lactose, raffinose, and stachyose. (D) Ratio of buffer removal for the three mixtures.

Finally, experiments with lactose as a disaccharide and stachyose as a tetrasaccharide were also conducted in 50/150 mM TRIS/NaCl (pH 7.4) as the best performing buffer system (Figure 8). The recovery rates are, as expected, higher for raffinose and stachyose compared to lactose (Figure 8A). Surprisingly, the yield for stachyose at a Q_DF_/Q_Feed_ ratio of 4:1 is only 55% compared to the 74.4% yield for raffinose at the same ratio (Figure 8A). For both raffinose and stachyose, a purity greater 95% could be achieved at Q_DF_/Q_Feed_ ratios of 4:1 and 6:1 (Figure 8C). The lower purity of lactose (92.1%) at a Q_DF_/Q_Feed_ ratio of 6:1 can be attributed to the significantly lower yield of 25.2%. The recovery rate for uridine is consistent across all three mixtures, suggesting that the type of saccharide present does not have a significant effect on uridine removal (Figure 8B). At lower flow rates of the diafiltration medium, buffer removal appears slightly higher for lactose compared to the two larger saccharides; however, this difference becomes negligible at higher flow rates, indicating that the molecular weight of the saccharide also has an insignificant effect on buffer removal (Figure 8D).

## 4 Conclusion

In this study, we demonstrated that the NFW nanofiltration membrane, with a molecular weight cut‐off between 300 and 500 Da, effectively separates the disaccharide lactose from the nucleoside uridine. This capability extends to the separation of larger oligosaccharides, such as raffinose and stachyose. We examined the impact of the transversal diafiltration medium stream on product yield within the module and found that higher product accumulation on the membrane surface, due to stronger convective forces, resulted in a higher concentration gradient and a stronger diffusion effect through the membrane, ultimately leading to lower yields. This effect was not evident in previous investigations of the module using ultrafiltration membranes [33, 34]. Implementation of a fluid guiding element serving as a static mixer provided a solution to this problem. It increased dispersion, disrupting the concentration polarization layer on the membrane and thereby reducing diffusive effects through the membrane and improving product yields. Screening the effect of different buffer systems, in which glycosyltransferases are active, on product purity and yield resulted in achieved purities greater than 95% while maintaining yields over 74%. Generally, it can be said that the module's performance improves with the molecular weight of the used saccharide. Furthermore, we were able to show the effect of the feed flow rate on the dispersion effect and the associated degree of buffer removal. From this, we were able to learn that the charge of the HEPES buffer at pH 7.5 significantly hinders the separation effect between saccharides and nucleotides in the SPNFDF operation mode, something which was not observed in the conventional SPTFF experiments.

When scaling up this nanofiltration process, one significant challenge arises from the risk of membrane damage under high operating pressures, which can lead to premature wear and tear and compromise overall performance. To address this issue while increasing flow rates, it is necessary to use modules with expanded middle compartments and larger membrane surface areas, thereby distributing pressure more evenly and mitigating the risk of damage. Another key consideration in this context is the choice of membrane configuration and associated cleaning strategies. Traditional cassette systems—often used in ultrafiltration—tend to be expensive and require elaborate cleaning procedures. Instead, employing cost‐effective flat‐sheet membranes simplifies both maintenance and replacement, as fouled membranes can be swapped out quickly and inexpensively. Furthermore, the use of small, 3D‐printed modules enables a straightforward “numbering‐up” strategy by running multiple units in parallel, an approach that can be extended to larger scaled‐up modules, such as the one already introduced in the past for UF/DF of proteins [34]. This modular setup not only lowers production and maintenance costs but also reduces downtime, providing a flexible and efficient solution for scaling up membrane‐based processes.

The results show that our continuous single‐pass nanofiltration/diafiltration module is a new, promising method for fast downstream processing of complex oligosaccharides. In addition, future optimization of the static mixer, as well as of the operating parameters and applied membrane type, should include the potential to further improve the obtained yields.

Further investigation of the influence of buffer concentration on the purification process, as well as the purification performance utilizing membranes with a higher MWCO, is warranted, as it should result in improved performance for larger oligosaccharides as well as for the separation of guanosine, which is a by‐product of enzymatic fucosylation reactions [57, 58]. A nanofiltration membrane with a higher MWCO should also improve buffer removal and the achieved purities in a HEPES system while maintaining high recovery rates for larger glycans such as HMOs and blood group antigen tetraose or pentose structures.

## Author Contributions

**Ulrich Thiele**: conceptualization, methodology, investigation, writing – original draft, writing – review and editing. **Tobias Kaloghlian**: investigation, data curation. **Jonas Wohlgemuth**: resources. **Gerald Brenner‐Weiß**: project administration, writing – review and editing. **André Tschöpe**: writing review and editing, supervision. **Matthias Franzreb**: writing – review and editing, supervision, funding acquisition. **Katharina Bleher**: supervision, writing – review and editing.

## Conflicts of Interest

The authors declare no conflict of interest

## Supporting information




**Supporting Information file 1**: elsc70055‐sup‐0001‐SuppMat.docx

## Data Availability

The data that support the findings of this study are available from the corresponding author upon reasonable request.
