# Supplementary material for: Fractionation of Oligosaccharide Nucleoside Mixtures by Single Pass Nano‐Diafiltration
Source: Eng Life Sci. 2025 Oct 13;25(10):e70055. doi: 10.1002/elsc.70055 (PMC12518163; doi:10.1002/elsc.70055)
Supplement: Supplementary file 1 — Supporting Information file 1: elsc70055‐sup‐0001‐SuppMat.docx [file ELSC-25-e70055-s001.docx]

**Fractionation of oligosaccharide nucleoside mixtures by single pass nano-diafiltration**

*Ulrich Thiele^a^, Tobias Kaloghlian^a^, Jonas Wohlgemuth ^a^, Gerald Brenner-Weiß ^a^, André Tschöpe ^a^, Matthias Franzreb ^a^ and Katharina Bleher*^a^*

*a)* Institute of Functional Interfaces, Karlsruhe Institute of Technology, Hermann-von-Helmholtz-Platz 1, 76344, Eggenstein-Leopoldshafen, Germany

# Supporting Information

Table of Contents

[Model reaction 3](#_Toc190871590)

[Conventional single-pass tangential flow filtration for membrane characterisation 4](#_Toc190871591)

[FIA-ESI-MS Configuration 4](#_Toc190871592)

[Calibration curves – Concentration 5](#_Toc190871593)

[Calibration curves - Conductivity 6](#_Toc190871595)

# Model reaction


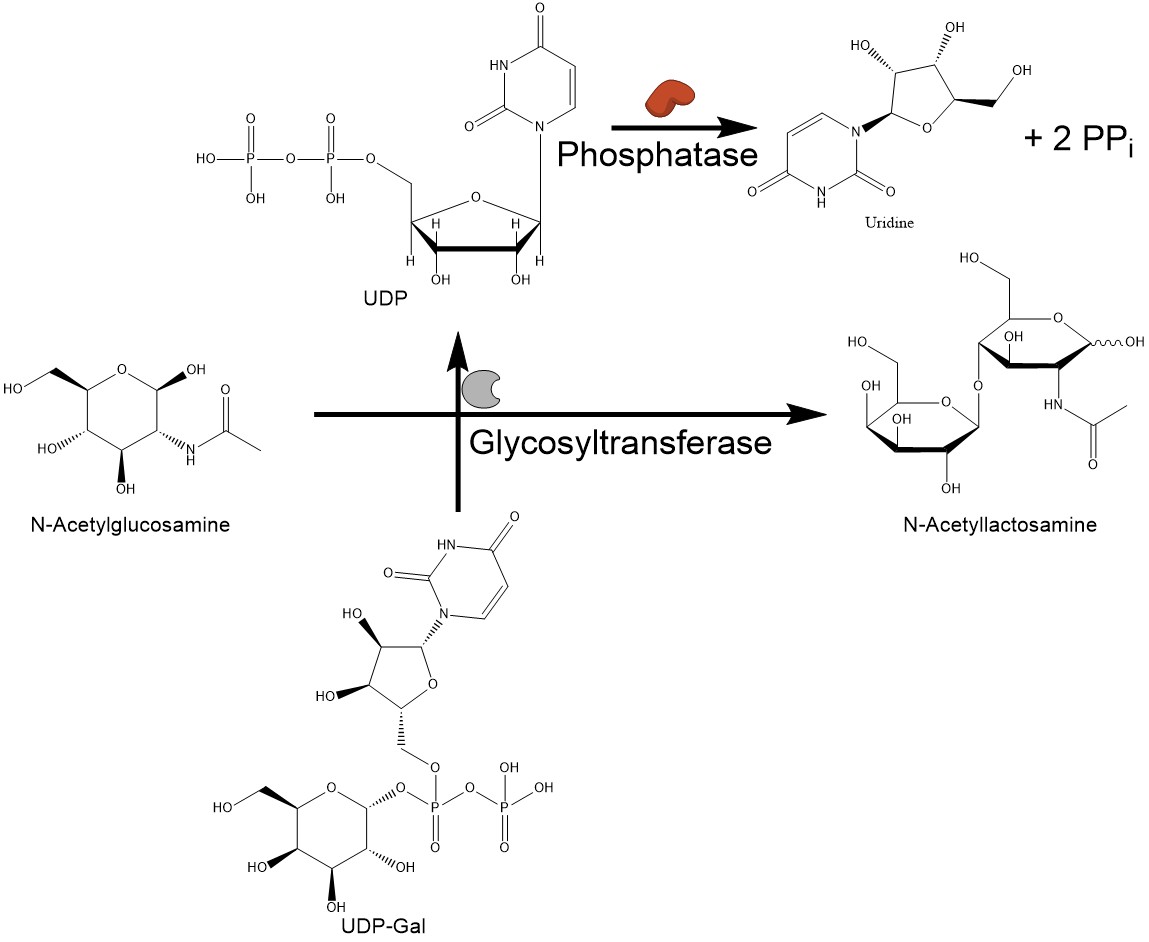


**Figure S1** Illustration of a model reaction catalyzed by a glycosyltransferase. It converts N-Acetylglucosamine to N-Acetyllactosamine by transferring a galactose monomer from UDP-Gal. The byproduct UDP formed in the process is then broken down by a phosphatase, resulting in a mixture of a disaccharide and the nucleoside uridine.

# Conventional single-pass tangential flow filtration for membrane characterisation


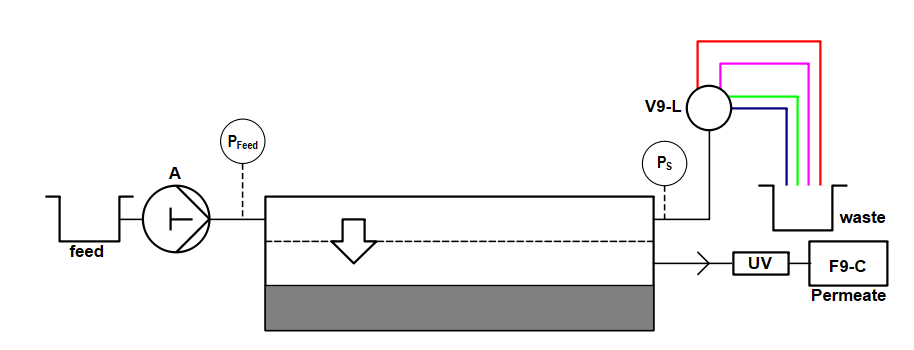


**Figure S2** Process scheme of the experimental setup for conventional single pass tangential flow filtration using an ÄKTA Pure 25 M. The 3D printed module was installed after pump A. A loop valve (V9-L) was used to switch between four capillaries with increasing length. Permeate samples were taken with a F9-C fraction collector. The grey lateral part can be seen in Figure.1B.

# FIA-ESI-MS Configuration

**Table S1** Operating conditions for FIA-ESI-MS in negative mode.

|  |  |  |  |  |
| --- | --- | --- | --- | --- |
| Curtain Gas |  |  |  | 30 psi |
| Ion source gas 1 |  |  |  | 25 psi |
| Ion souce gas 2 |  |  |  | 50 psi |
| Temperature |  |  |  | 450 °C |
| Ion spray voltage |  |  |  | -4500 V |

**Table S2** Analytes are listed with the fragment ions used for quantification, as well as the DP (declustering potential) and CE (collision energy) values employed.

| Analyte | Precursor-ion | Fragment-Ion | DP | CE |
| --- | --- | --- | --- | --- |
| D-Lactose | 341.10 | 179.05 | -25.0 | -10.0 |
| D-Raffinose | 503.20 | 179.05 | -75.0 | -42.5 |
| D-Stachyose | 665.20 | 383.12 | -75.0 | -42.5 |
| Uridine | 243.06 | 111.02 | -30.0 | -15.0 |
| 1-^13^C D-Glucose | 180.06 | 119.03 | -25.0 | -10.0 |
|  |  |  |  |  |

# Calibration curves – Concentration

Calibrations between 0 – 80 ng/mL were prepared for FIA-ESI-MS (Table S2).

**Table S3** Stock solutions were prepared by dissolving the analytes in a 50:50 mixture of ACN and water to achieve a concentration of 1 mg/mL. These solutions were subsequently diluted to 1000 ng/mL for the analytes and to 10000 ng/mL for the internal standard. For each calibration mixture, 10 µL of the internal standard was added, resulting in a final concentration of 100 ng/mL ^13^C-D-Glucose

| Final Concentration of Analytes  [ng/mL] | Added solvent (50:50 ACN/H_2_O)  [µL] | Added Stock solution of Analytes (1000 ng Stock) [µL] | Added Volume of ISTD 1-^13^C-D-Glucose [µL] | Total Volume [µL] |
| --- | --- | --- | --- | --- |
| 0 | 990 | 0 | 10 | 1000 |
| 2.5 | 987.5 | 2.5 | 10 | 1000 |
| 5 | 985 | 5 | 10 | 1000 |
| 10 | 980 | 10 | 10 | 1000 |
| 20 | 970 | 20 | 10 | 1000 |
| 30 | 960 | 30 | 10 | 1000 |
| 40 | 950 | 40 | 10 | 1000 |
| 50 | 940 | 50 | 10 | 1000 |
| 60 | 930 | 60 | 10 | 1000 |
| 70 | 920 | 70 | 10 | 1000 |
| 80 | 910 | 80 | 10 | 1000 |

#

**Figure S3** A: Calibration for lactose (y=0.0099x; R^2^=0.9985). B: Calibration for raffinose (y=0.0025x; R^2^=0.995). C: Calibration for stachyose (y=0.0068x; R^2^=0.995). D: Calibration for uridine (y=0.1762x; R^2^=0.9994).

# Calibration curves - Conductivity

**Figure S4** A: Calibration for the exchange of 100/25 mM HEPES/KCl to ddH_2_O (y=7.64-0.0762x; R^2^=0.9981). B: Calibration for the exchange of 50/150 mM TRIS/NaCl to ddH_2_O (y=21.66-0.216x; R^2^=0.9983). C: Calibration for the exchange of 100 mM MOPS to ddH_2_O (y=5.03-0.0499x; R^2^=0.9978).
